# Berberine restrains the expansion of colorectal cancer organoids by blocking cell cycle progression and reducing lipid synthesis

**DOI:** 10.3389/fimmu.2026.1855151

**Published:** 2026-07-10

**Authors:** Hefei Tian, Tianshuo Zhao, Banghui Liu, Yujun Huang, Xi Wang, Zhenni Xu, Lingxiao Huang, Xudan Lei, Mingyue Qu, Qiongying Hu, Dengqun Liu

**Affiliations:** 1 School of Basic Medical Sciences, Chengdu University of Traditional Chinese Medicine, Chengdu, China; 2 Precision Radiation in Oncology Key Laboratory of Sichuan Province, Department of Experimental Research, Sichuan Cancer Hospital & Institute, Sichuan Provincial Engineering Research Center for Tumor Organoids and Clinical Transformation, Sichuan Clinical Research Center for Cancer, Sichuan Cancer Center, School of Medicine, University of Electronic Science and Technology of China, Chengdu, China; 3 The People’s Liberation Army (PLA) Rocket Force Characteristic Medical Center, Beijing, China; 4 Department of Laboratory Medicine, Hospital of Chengdu University of Traditional Chinese Medicine, Chengdu, Sichuan, China

**Keywords:** berberine, cell cycle, colorectal cancer, lipid metabolism, organoid, RNA-Seq

## Abstract

**Introduction:**

Among all the most prevalent malignant gastrointestinal cancers, colorectal cancer (CRC) occurs frequently in all the populations around the world. Despite remarkable advances in related research, substantial obstacles persist in the prevention and treatment of this malignancy, including safety concerns, adverse side effects, and tumor recurrence. Natural plant-derived compounds have increasingly attracted the attention of researchers in cancer research. Among these compounds, berberine (BBR) is a natural plant substance with multiple functions, which is extracted from *Coptis Chinensis* that possesses strong antitumor potential. Here we aim to investigate the effects and underlying mechanisms of BBR on CRC using several organoid models developed in our laboratory.

**Methods:**

We firstly established CRC organoid models derived from KPC transgenic mice and Caco-2 cell line. CRC organoids were treated with different doses of BBR. The morphological characteristics, proliferation, ROS, apoptosis, and cell cycle were carefully evaluated. RNA-Seq assay, epithelial permeability, and lipid probes were also used to examine the underlying molecular mechanisms of BBR on CRC organoids.

**Results:**

BBR exhibited no harmful impact on healthy colonic tissues. However, BBR significantly inhibited the growth of both KPC organoids and Caco-2 organoids either at the early formation stage or after the maturity. BBR greatly decreased the ratio of Ki67 and EdU positive cells in CRC organoids and increased the level of ROS in organoids. RNA-Seq data implicated that BBR exerted direct cytotoxic effects on CRC organoids by inducing cell cycle arrest and breaking down the gap junctions. Immunohistochemical (IHC) staining identified the consistent changes of cell cycle arrest and dysfunction of epithelial barrier. FD4 staining showed increased epithelial permeability. Finally, LD540 probes and qPCR identified that BBR significantly blocked the lipid synthesis in CRC organoids.

**Conclusions:**

In summary, our study identifies that BBR may suppress the malignant phenotype of CRC organoids via multiple mechanisms, including blocking cell cycle progression and disrupting lipid metabolism.

## 1 Introduction

Colorectal cancer (CRC) is still a main global health burden, which ranks the third most prevalent malignancy and the second leading cause of cancer-related death worldwide (1, 2). Unfortunately, its incidence keeps rising across both high- and middle-income nations, including China, alongside a concerning surge in early-onset cases (diagnosed before age 50). Such a shifting epidemiological landscape thus attracts more attentions of both researchers and clinicians. While current therapies, including surgery, chemotherapy, immunotherapy, and targeted agents, have improved outcomes, their efficacy is persistently undermined by high recurrence rates, heterogeneous responses, and drug resistance, creating an urgent need for novel therapeutic avenues. Phytomedicines and plant-derived compounds have emerged as compelling candidates in this context. Many traditional Chinese herbs and their bioactive constituents exhibit potent anti-tumor activities comparable to conventional chemotherapeutics, offering a promising, multi-targeted strategy for CRC therapy.

Berberine (BBR) has a chemical structure of C_20_H_18_NO_4_^+^, and it is a quaternary ammonium alkaloid, which is principally isolated and purified from natural plants belonging to the *Berberidaceae*, *Ranunculaceae*, and *Rutaceae* families. The medicinal value of BBR-containing plants, particularly *Coptis chinensis*, was first documented around year 200 AD in the Divine Farmer’s Classic of Materia Medica. And till now, BBR has been recorded for its use in treating diabetes, gastroenteritis, and polycystic ovary syndrome, etc (3, 4). In addition, BBR has long been widely used as an over-the-counter medication for treating clinical intestinal disorders and bacterial diarrhea. Recently, we have reported that BBR is able to modulate the viability of intestinal stem cell (ISCs) and the dysfunction of salivary secretion in physiological and irradiated mice (5, 6). Beyond the anti-inflammatory, antibacterial, antiarrhythmic, hypoglycemic, and hypotensive effects, the anticancer capability of BBR has garnered significant attention (7).

Increasing evidence indicates that BBR could apparently block the growth of many cancers, including gastric cancer, lung cancer, breast cancer, and ovarian cancers, etc. Of them, the efficacy of BBR against CRC is being particularly well-recognized. Recent clinical studies confirm that BBR could effectively suppress the recurrence of colorectal cancer. Meanwhile, BBR suppresses Wnt pathway activity through multiple mechanisms, which is a critical pathway during the development of colorectal cancer. In this cascade, BBR could interrupt the binding between Wnt molecules and their receptor, impair β-Catenin expression levels and the formation of the β-Catenin/transcription factor (TCF) complex. Additionally, BBR is capable to arrest cell cycle propagation by modulating cyclin D1, which is an important protein for the transition from G1 to S phase. BBR primarily induces apoptosis through endogenous mitochondrial apoptosis pathways by activating mitochondrial signaling via ROS mediated signaling pathways such as JNK/p38 MAPK, PKC, and ERK (8). BBR also directly damages mitochondrial membranes, leading to apoptosis (9). BBR can induce a series of characteristic morphological alterations associated with autophagy in cancer cells, and the involvement of autophagic proteins, for example, Bcl-2 and Beclin-1, confirms the influences of BBR on autophagy process (10). Due to its multiple targets, minimal side effects, and low cost, BBR is emerging as a powerful natural plant compound for the management of colon cancer.

Gut barrier function and lipid metabolism are essential to the composition of intestinal epithelium. BBR inhibits Th1 and Th17 cell proliferation and suppresses NF-κB signaling while limiting pro-inflammatory factors, such as TNF-α, IFN-γ (11, 12). It also modulates opioid receptors to suppress intestinal edema, exerts antisecretory activity in the small intestine, prevents the damage to epithelial barrier, and restrains the translocation of gut microbiota (13). Therefore, BBR is recognized as a promising drug for ulcerative colitis (14, 15). BBR could inhibit tumor invasion and metastasis via inducing p53, regulating ROS, suppressing tumor cell proliferation, and suppressing epithelial-mesenchymal transition (EMT) (16, 17). Previously, BBR has been shown to inhibit the progression of CRC by restoring gut microbiota. BBR directly binds to the G-quadruplex structure of HIF-1α and inhibit HIF-1α protein expression by suppressing mTOR phosphorylation (18). Furthermore, BBR treatment reduced D-lactic acid levels in tumor tissues by downregulating HIF-1α and upregulating ornithine decarboxylase antagonist 1 (OAZ1) (19). Berberine also inhibits the cell proliferation of colon cancer by regulating the β-Catenin pathway and enhancing the activity of ubiquitin ligase CbI, while enhancing ROS production and activating JNK/p38 MAPK pathway to induce apoptosis (20, 21).

However, most of the previous investigations of BBR’s impact on CRC have primarily performed by traditional two-dimensional (2D) adherent culture models, which are nonidentical to *in vivo* structures. Organoids are three-dimensional (3D) culture systems, and they are composed by heterogenous cellularity. Therefore, they can mimic the structural and functional characteristics of cancer. And they can more really reflect the biological responses of different treatments. In particular, organoid culture technology preserves tumor tissue heterogeneity and cell-cell interactions, providing a more physiologically relevant *in vitro* model for drug research (2). Here, we aim to explore the direct pharmacological effects of BBR on CRC using cancer organoids and explore the underlying mechanisms.

## 2 Materials and methods

### 2.1 Animals

Male C57BL/6 mice aged 6–8 weeks and weighing 18–22 g were purchased from HFK Biotechnology Co., Ltd. (Beijing, China). B6.Cg-*Kras^tm4Tyj^ Apc^tm1Tno^* Tg(CDX2-cre/ERT2)752Erf/MaraJ were introduced from Jackson Laboratory (Jax ID: 035169, Bar Harbor, ME, USA) (22), and abbreviated as KPC mice in this study. Prior to experimental use, all mice were acclimated to laboratory conditions for 3 days. Animals were housed in a specific pathogen-free (SPF) facility under a 12 h light/12 h dark cycle, with free access to standard chow and sterile water. All animal procedures were performed in accordance with the NIH Guide for the Care and Use of Laboratory Animals (8^th^ edition). The experimental protocol was reviewed and approved by the Ethics Committee of Sichuan Cancer Hospital (No. SCCHEC-04-2024-033), complying with the current regulations on laboratory animal welfare.

### 2.2 BBR treatment for mice

Berberine hydrochloride (BBR, HY-18258, MCE, China) was prepared by dissolving in DMSO to make the stocking solution, and the working solution was freshly prepared before administration. C57BL/6 mice were divided into two groups: control group and BBR-treated group. In the BBR group, mice were treated by intraperitoneal (i.p) injection of BBR. The dose of BBR was 5 mg/kg, and mice were injected every day for 6 days before analysis. Meanwhile, mice in the control group were given the i.p injections of the same volume of PBS over the same 6-day period.

### 2.3 Induction of *in vivo* CRC model from KPC mice

KPC mice were bred in an SPF environment, and their offspring were subjected to genotyping before experimental use. Genotyping was performed according to the protocol provided by The Jackson Laboratory. Male offspring with the correct genotype were selected for experiments at 6–8 weeks of age. Briefly, tamoxifen (T5648, Sigma-Aldrich, USA) was freshly dissolved in sunflower oil for the dose of 10 mg/mL. Mice were intraperitoneally injected at a dose of 2 mg per 20 g body weight for 3 consecutive days to activate Cre recombinase. Mice were euthanized on day 8 after the first injection, and colons were collected for subsequent histological analysis and organoid culture.

### 2.4 Crypt isolation and organoid culture

Pentobarbital sodium was dissolved in normal saline for 1% (w/v) concentration, and mice were deeply anesthetized at the dose of 100 mg/kg by intraperitoneal (i.p) injection of 0.2 mL pentobarbital sodium solution per 20 g weight. After deep anesthesia, healthy or KPC mice were sacrificed by cervical dislocation, and fresh colonic tissues were quickly collected. Then the adipose tissues were carefully removed. The contents in colons were flushed by ice-cold PBS. Colon was longitudinally opened and cut into 3~5 mm pieces. Tissue segments were extensively rinsed and vigorously washed in cold PBS. Then tissues were incubated in chelation buffer containing 5 mM EDTA (#25300096, Invitrogen) and 1% penicillin/streptomycin on ice for 45 min. Colonic crypts were mechanically dissociated by frequent pipetting. Isolated crypts were enriched by centrifugation at 800 rpm for 3 minutes. Pelleted crypts were resuspended in Matrigel (#354230, Corning, USA), and they were seeded into 96-well plates. Organoids were cultured as previously described (23). Healthy and KPC colonic organoids were respectively treated by BBR at the doses from 12.5 μg/mL to 100 μg/mL as indicated.

### 2.5 Cell culture and organoid formation assay

Human colorectal cancer (CRC) cell line Caco-2 was introduced from the Cell Bank of the Chinese Academy of Sciences (Shanghai, China) and maintained by our institute. Cells were cultured in the corresponding basal culture medium and supplemented with 10% fetal bovine serum (FBS) and 1% penicillin/streptomycin solution (Beyotime, China). For organoid culture, Caco-2 cells were resuspended in Matrigel (Corning, USA) and planted into 96-well plates with the density of 500 cells per 10 μL Matrigel to facilitate hollow organoid formation (24). Upon the maturation of organoids, berberine (BBR) was introduced into the culture medium at the doses of 12.5 μg/mL, 25 μg/mL, 50 μg/mL, and 100 μg/mL, respectively. Subsequent analyses were performed to evaluate the morphological changes, proliferative capacity, and intracellular reactive oxygen species (ROS) levels of the organoids.

### 2.6 Detection of cell proliferation and ROS level

Cell proliferation in CRC organoids was assessed using the EdU assay. Briefly, KPC and Caco-2 organoids were cultured for 3 days, followed by treatment with different concentrations of BBR overnight till 24 h. The organoids were then incubated with EdU (10 μM, ST067, Beyotime, China) for 2 hours and then fixed in 4% paraformaldehyde (PFA) for 30 min. EdU staining was performed by BeyoClick™ EdU-488 Kit (C0071S, Beyotime, China) following the protocols provided by manufacturer. Intracellular ROS levels after BBR treatment were detected using a dihydroethidium (DHE) probe (S0063, Beyotime, China). Briefly, DMSO dissolved DHE probe was loaded into cell culture medium at a final dose of 5 μM. Organoids were then incubated with the DHE probe for 30 min and observed. Fluorescent signals were captured using the corresponding filter channels.

### 2.7 Hoechst 33342/PI staining assay

Mature KPC or Caco-2 organoids were cultured and treated with different concentrations of BBR for 24 hours. After removing the BBR-containing culture medium, CRC organoids were washed and incubated in fresh culture medium with Propidium Iodide (PI, ST511, Beyotime, China) and Hoechst 33342 (C1027, Beyotime, China) for 15 minutes. After three washes with PBS, fluorescent images were taken by M5000 microscope (Thermofisher, USA).

### 2.8 Cellular lipid staining assay

Lipid in the CRC organoid cells was demonstrated using Lipid Droplet Red Fluorescence Assay Kit with LD540 (C2050, Beyotime, China) in accordance with the manufacture’s protocol. Briefly, BBR was used to treat KPC and Caco-2 organoids for 24 hours. The staining buffer was freshly prepared containing LD540 (1000x), Hoechst 33342, and assay buffer following the provider’s instruction. The final concentration of LD540 was 0.5 μg/mL. Organoids were incubated with LD540 staining buffer for 30 min in the incubator. After that, the lipid droplets within CRC organoids were observed, and the images were capture with Cytation 5 (Agilent Technologies, USA).

### 2.9 Apoptosis inhibitor treatment

KPC organoids were cultured for 3 days to form mature organoids. Subsequently, the apoptosis rescue experiment was performed. Briefly, the organoids were divided into two groups: the control group and the combined treatment group with BBR and apoptosis inhibitor. Mature KPC organoids were pretreated with 10 μM pan-caspase inhibitor Z-VAD-FMK (HY-16658, MCE, China) for 1 h. Different doses of BBR were then added to culture medium, and the organoids were continuously incubated with Z-VAD-FMK during the experiment. An equal volume of dimethyl sulfoxide (DMSO) was injected for the control group to eliminate the interference caused by the solvent itself.

### 2.10 Evaluation of organoid permeability

The permeability of CRC organoids was evaluated by FITC-Dextran (FD4) assay. Briefly, KPC and Caco-2 organoids were cultured in the 96-well plate for 3 days, and BBR was loaded at the concentrations of 12.5 μg/mL, 25 μg/mL, 50 μg/mL, and 100 μg/mL respectively. After a 24h culture, BBR was removed, and FD4 (Sigma Aldrich, USA) was diluted with fresh culture medium for 1 μg/mL. Organoids were incubated with FD4 for 30 min, and then the additional FD4 was thoroughly washed by PBS, and the FD4 signals within organoids was observed by Cytation 5 under 488nm channel as previously described.

### 2.11 Organoid harvest and staining

KPC and Caco-2 organoids were placed in cell recovery solution (CRS, #354253, Corning, USA), incubated on ice for 30 minutes, and then collected separately. After Matrigel dissociation, the organoid suspension was gently centrifuged at the speed of 800 round per minute for 3 minutes. After removing the supernatant, the organoid pellet was fixed in pre-cooled 4% paraformaldehyde (PAF, #BL539A, Biosharp) at 4 °C for 30 minutes. The fixed organoids were embedded and sectioned according to the standard protocol of our laboratory. Conventionally, 4-μm-thick sections were deparaffinized in xylene, rehydrated through a graded ethanol series. Antigen retrieval was performed by boiling in TRIS-EDTA antigen retrieval solution (#BL618A, Biosharp) for 15 minutes. Tissue sections were incubated with the blocking buffer containing 1% bovine serum albumin (A7906, Sigma-Aldrich) and 0.5% Tween X-100 for 30 minutes, and tissues were incubated with diluted primary antibodies overnight at 4 °C. Details for antibody preparation were listed in **Supplementary Table 1**. Immunohistochemistry (IHC) staining was developed using HRP-linked secondary antibodies, and the DAB kit was used for visualization (ZSBio, China). During immunofluorescent (IF) staining, highly cross-adsorbed donkey anti-rabbit/mouse IgG (H+L) antibodies conjugated to AlexFluo™ 488 or 594 (ThermoFisher, USA) were employed, and cell nuclei were stained with DAPI (Vector Lab, California, USA). Images were acquired using an Olympus BX53 microscope, a Zeiss Axio Observer equipped with Apotome3, or the Olympus confocal microscope.

### 2.12 Transmission electron microscopy

KPC organoids were assigned to control and BBR-treated groups. After 24 h of treatment, fresh KPC organoids were harvested from Matrigel, enriched by low-speed centrifugation, and processed for TEM as previously reported (25). Briefly, organoid pellets were rapidly processed for fixation for 1 h in cold 0.1 M Sodium Cacodylate-HCl buffer (pH 7.4) with 4% PFA and 1% glutaraldehyde, then washed three times (15 min each) in 0.1 M cacodylate buffer supplemented with 0.1 M glycine. Samples were postfixed in 1% osmium tetroxide (0.1 M cacodylate buffer), dehydrated via a methanol gradient to propylene oxide, and embedded in epoxy resin. Ultrathin sections were mounted on grids and examined/photographed by TEM (Tecnai-10, Philips, Amsterdam, Netherlands) at 100 kV accelerating voltage.

### 2.13 RNA extraction and qRT-qPCR

RNA samples were isolated from BBR-treated Caco-2 organoids (24-hour treatment duration) using RNAiso Plus reagent (9109, TaKaRa, Japan). RNA concentration was measured with a NanoDrop2000 (ThermoFisher, USA). Hifair II 1st Strand cDNA Synthesis Super Mix (11137ES60, YEASEN, China) was utilized for reverse transcription, while qPCR assays were performed on a Bio-Rad C1000 thermal cycler (Bio-Rad, USA) with SYBR Green PCR Master Mix (11203ES08, YEASEN, China). The expression levels of target genes were normalized to β-Actin, and then determined by the 2*^-ΔΔCt^* formula. Finally, the sequences of each primer pair are provided in the **Supplementary Materials** (**Supplementary Table 2**).

### 2.14 RNA-Seq and data analysis

The extraction and evaluation of RNA samples from KPC organoids were performed as described above. RNA-seq was performed by Shanghai OE Biotech Co., Ltd. Briefly, the integrity of RNA quality was assessed by Agilent 2100 (Agilent Technologies, USA). The library was prepared with the VAHTS Universal V6 RNA-seq Library Prep Kit and sequenced on the Illumina NovaSeq 6000 platform, with sequencing data analyzed by OE Biotech Co., Ltd. Gene FPKM values were determined, and the counts of gene read were determined by HTSeq-count. Principal components analysis (PCA) was calculated using R (v 3.2.0) to assess sample biological duplication. Differential expression genes were determined by DESeq2, with significantly differentially expressed genes (DEGs) defined by Q < 0.05, and the fold change was more than 2 times or less than 0.5 times. DEG enrichment (GO, KEGG pathway, Reactome, WikiPathways) were conducted using R (v 3.2.0), and Gene Set Enrichment Analysis (GSEA) was conducted with GSEA analysis software.

### 2.15 Statistics

All data in this study are presented as mean ± standard deviation (SD), with statistical analysis performed using GraphPad Prism 9 (Graphpad Software, USA). Two-tailed unpaired Student’s t-test was used for two-group comparisons, and one-way ANOVA for three or more groups. Statistical significance was defined as *: P < 0.05, **: P < 0.01, ***: P < 0.001, ****: P < 0.0001; P > 0.05 was considered nonsignificant (n.s).

## 3 Results

### 3.1 BBR exhibits no apparent toxicity on colon tissues and organoids in physiology

**Figure 1:**
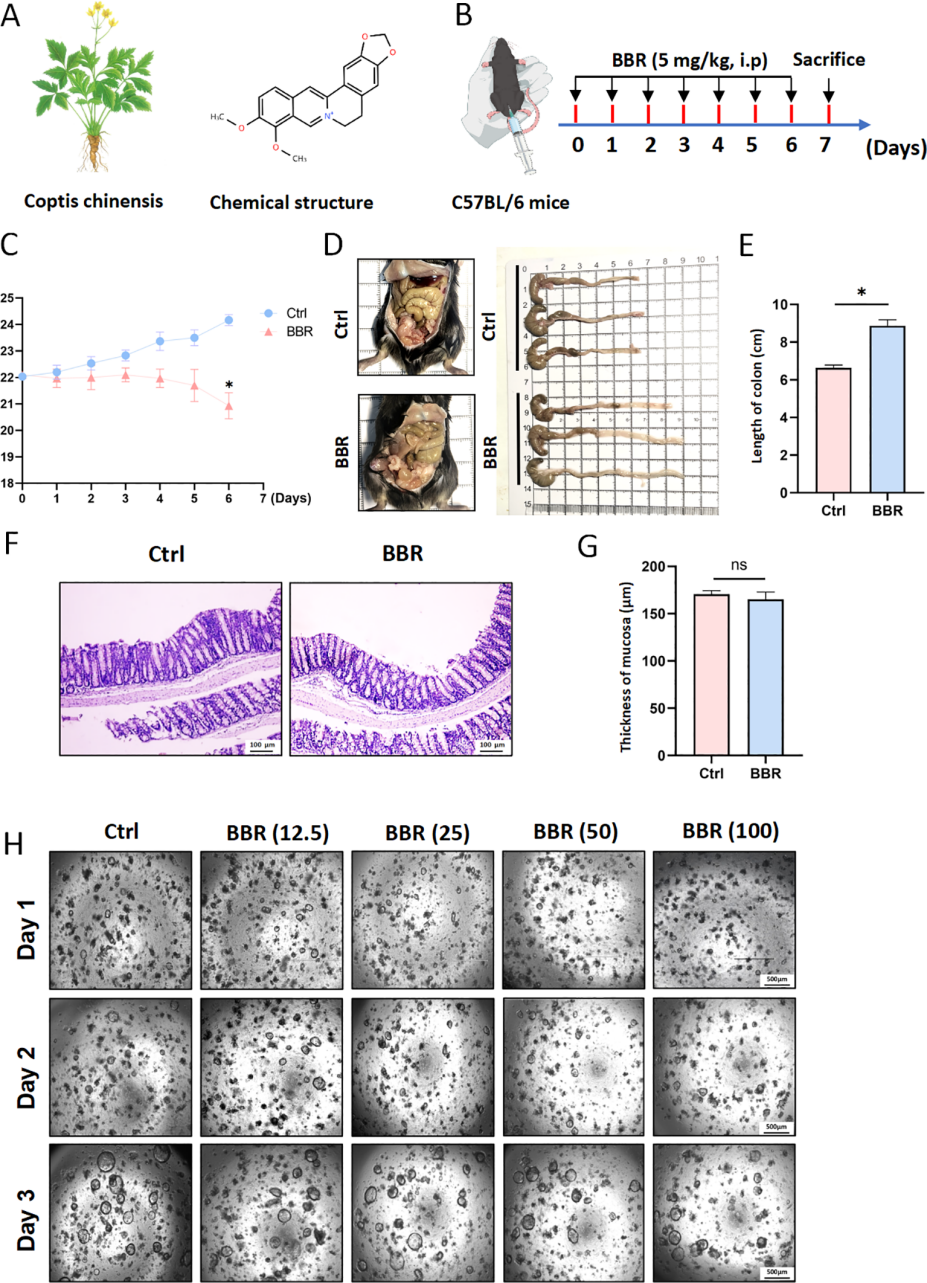
BBR shows no significant toxicity on colon morphology and organoid in physiological condition. **(A)** *Coptis chinensis* and BBR’s chemical structure. **(B)** Experimental design for the BBR treatment of healthy C57BL/6 mice. Mice received daily i.p injection of 5 mg/kg BBR for 6 continuous days and tissues were collected at day 7. **(C)** The curve of body weight changes between control group and BBR group (n=3 per group). **(D)** Representative abdominal appearance and macroscopic image of colons in different groups. **(E)** Statistical analysis for the length of colons (n=3 per group). **(F)** Representative H&E staining of colon tissues in mice between control group and BBR group. Scale bar: 100 μm. **(G)** Quantitative comparison for the thickness of colonic mucosal layer (n=3 per group). **(H)** Colon organoids from healthy mice were treated by different doses of BBR ranging from 12.5 μg/mL to 100 μg/mL. Scale bar: 500 μm. *P < 0.05, *n.s*, not significant.

At the initial stage of this study, we first evaluated the effects of BBR on healthy colonic tissues under physiology. Healthy C57BL/6 mice were intraperitoneally injected with 5 mg/kg BBR every day for 6 days (**Figures 1A, B**). It was found that BBR caused a light decrease of body weight (**Figure 1C**). At day 7, there were no obvious changes the appearance of gastrointestinal (GI) tract (**Figure 1D**). However, the length of colons was significantly increased (**Figure 1E**), which was similar to small intestines we have previously reported (5). We further examined the histology of colonic epithelium by hematoxylin-eosin (H&E) staining, and no great difference was observed from H&E images (**Figure 1F**), and the statistical result also showed similar mucosal thickness (**Figure 1G**). Since BBR might act on different cells (eg. epithelial cells, immune cells) *in vivo*, we also evaluated whether BBR could harm healthy colonic organoids *in vitro*. It was observed that even with the increasing of concentrations from 12.5 μg/mL to 100 μg/mL BBR exhibited no significant injury to healthy colonic organoids (**Figure 1H**). Therefore, it is confirmed that BBR exhibits no significant toxic effects on healthy colonic tissues and organoids at the doses adopted in this study.

### 3.2 Early BBR treatment limits the formation and growth of CRC organoids

**Figure 2:**
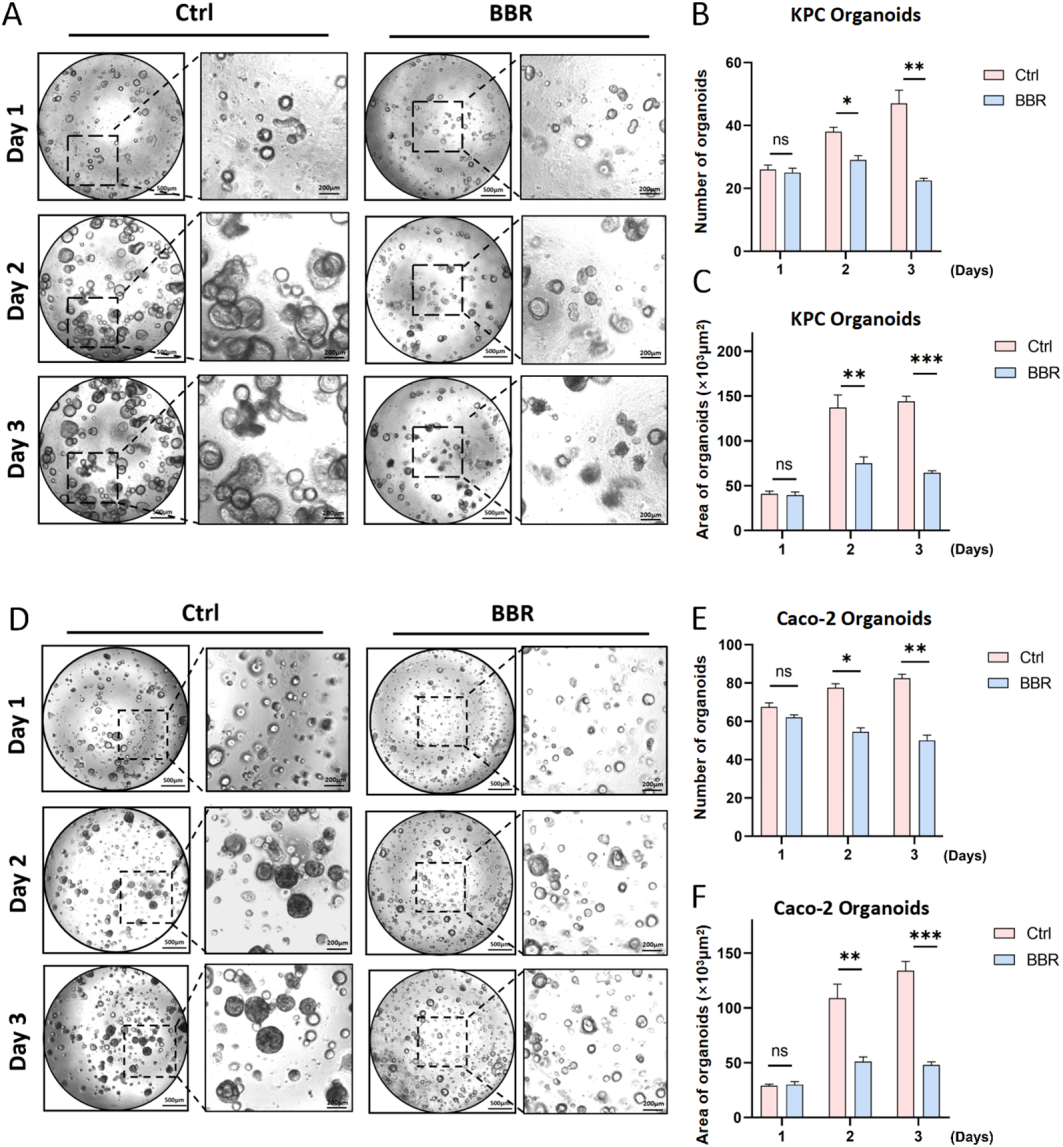
BBR inhibits the expansion of CRC organoids at the early growing stage. **(A)** Morphological images of KPC organoids in control group and BBR (25 μg/mL) group at Day 1, Day 2, and Day 3. Scale bar: 500 μm or 200 μm as indicated. **(B)** Quantitative analysis for the number of KPC organoid at indicated time points. **(C)** Statistical analysis for the surface area of KPC organoids at different time points in control group and BBR-treated group. **(D)** Representative images of Caco-2 organoids taken at Day 1, Day 2, and Day 3 after the treatment of 25 μg/mL BBR. Scale bar: 500 μm or 200 μm as indicated. **(E)** Statistical results for the number of Caco-2 organoids after BBR treatment. **(F)** Statistical analysis for the surface area of Caco-2 organoids at the indicated time points. *P < 0.05, **P < 0.01, ***P < 0.001, *n.s*, not significant.

As mentioned above, BBR has exhibited multiple biological effects on colorectal (CRC) cancer, and here we mainly aimed to investigate its influences on CRC organoids. Therefore, we established different CRC organoid models from mouse tissues and human cell lines. Firstly, we cultured CRC organoids from KPC mice. After tamoxifen induction, KPC mice could develop colorectal cancer within few days. We isolated the colonic crypts and cultured KPC organoids. Using KPC organoids, we screened different doses of BBR on the growth of CRC organoids at the early growth stage. It was observed that BBR treatment could apparently inhibit the initial growth of KPC organoids (**Supplementary Figure S1A**), then we used 25 μg/mL BBR to treat KPC organoids. The results showed opposite growth characteristics of KPC organoids between control group and BBR group (**Figure 2A**). Both the number and size of KPC organoids in BBR group were significantly decreased at day 2 and day 3 compared with control group (**Figures 2B, C**). Meanwhile, BBR could apparently inhibited the formation of Caco-2 organoids with a dose dependent pattern (**Supplementary Figure S1B**). A at dose of 25 μg/mL BBR also significantly decreased the number and size of Caco-2 organoids (**Figures 2D-F**). In addition, we cultured another CRC organoids from human HCT-116 cells and found that BBR could effectively block the formation and growth of HCT-116 spheres (**Supplementary Figure S1C**). Collectively, we find BBR is capable to effectively suppresses the formation and growth of different CRC organoids during the early culture stage.

### 3.3 BBR causes significant impairments to the mature CRC organoids

**Figure 3:**
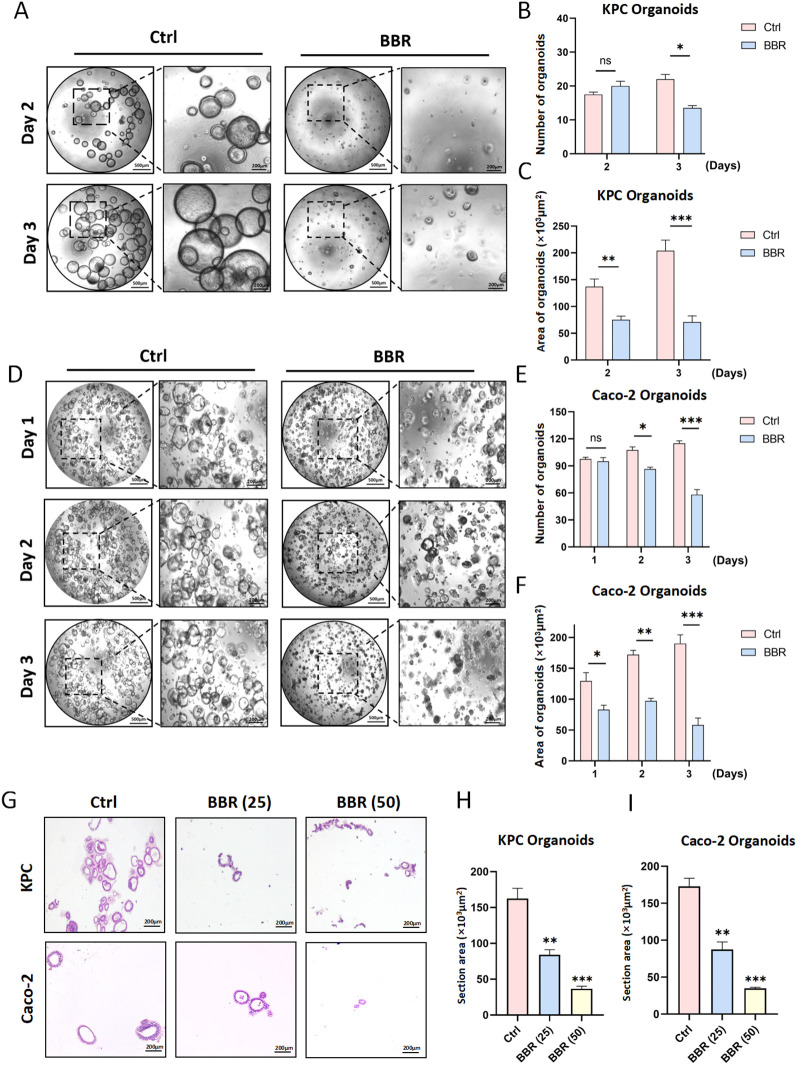
BBR effectively impairs the mature CRC organoids. **(A)** Comparison of representative appearance of mature KPC organoids in control group and 25 μg/mL BBR-treated group at Day 2and Day 3. Scale bar: 500 μm in low power images and 200 μm in high power images. **(B)** Quantitative analysis of the number of mature KPC organoids at Day 2 and Day 3 after BBR treatment. **(C)** Statistical results for the surface area of mature KPC organoids after BBR treatment. **(D)** Representative morphology of mature Caco-2 organoids under the same BBR treatment (25 μg/mL). **(E)** The quantitative results for the amount of mature Caco-2 organoids at the indicated time after BBR treatment. **(F)** Statistical analysis for the area of mature Caco-2 organoids. **(G)** H&E images of mature KPC and Caco-2 organoids at 36 h in control group and BBR-treated groups (25 μg/mL, 50 μg/mL). Scale bar: 200 μm. **(H)** Quantitative results for the section area of mature KPC organoids in each group. **(I)** Statistical analysis for the section area of Caco-2 organoids in control group and BBR-treated group. *P < 0.05, **P < 0.01, ***P < 0.001, *n.s*, not significant as compared to the control group.

Due to the previous evidence that BBR effectively inhibits the early growth of colon cancer organoids, we infer that BBR might also impair the mature CRC organoids *in vitro*. Therefore, we cultured KPC and Caco-2 organoids to validate this hypothesis. Interestingly, it was observed that mature KPC organoids were sensitive to the BBR treatment (**Supplementary Figure S2A**). As shown in the enlarged images, KPC organoids grew quickly in the control group (**Figure 3A**), but both the number and the area of KPC organoids at Day 2 and Day 3 after 25 BBR treatment were apparently decreased in comparison to the vehicle treated organoids (**Figures 3B, C**). When we also treated Caco-2 organoids with the same regimen, it was found that the mature organoids were seriously blocked (**Supplementary Figure S2B**), and with the extension of treatment fewer mature Caco-2 organoids were survived under the pressure of BBR (25μg/mL) (**Figure 3D**). Statistical analysis showed the identical results according to the number and area of Caco-2 organoids (**Figures 3E, F**). Moreover, KPC and Caco-2 organoids in control group and BBR-treated groups were embedded in paraffin. H&E staining images also showed smaller size of these two organoids in 36-hour BBR treatment compared to their normal mature counterparts (**Figures 3G-I**). So, our results clearly show that while BBR blocks the growth of CRC at the early stage, it can also result in significant impairment to the mature CRC organoids, indicating an important and strong antitumor activity of BBR for colorectal cancer.

### 3.4 BBR decreases cell proliferative level and blocks DNA replication in CRC organoids

**Figure 4:**
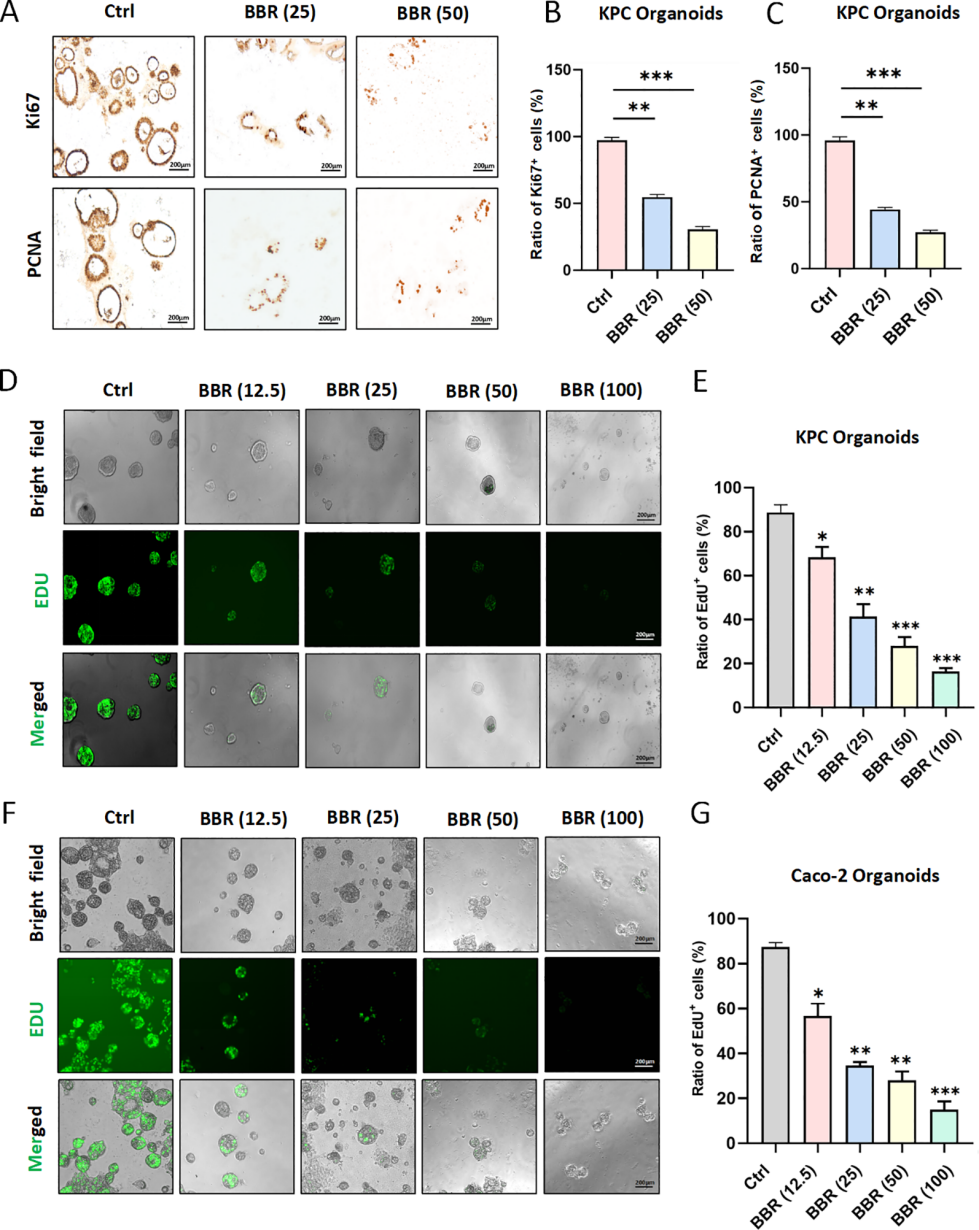
BBR limits cell proliferation and DNA replication activity in CRC organoids. **(A)** Representative images of IHC staining against Ki67 and PCNA positive cells within KPC organoids at 36 h in control group and BBR-treated groups (25 μg/mL, 50 μg/mL). Scale bar: 200 μm. **(B, C)** Quantitative analysis for the values of Ki67-positive and PCNA-positive cell proportions in different groups. **(D)** EdU staining images of KPC organoids at 24 h after gradient BBR treatments (12.5 μg/mL, 25 μg/mL, 50 μg/mL, and 100 μg/mL). Scale bar: 200 μm. **(E)** Statistical analysis for the ratio of EdU-positive cells in KPC organoids. **(F)** Representative images of EdU staining in Caco-2 organoids with the same treatments. Scale bar: 200 μm. **(G)** Quantitative analysis of EdU-positive cells in Caco-2 organoids after different BBR treatments. *P < 0.05, **P < 0.01, ***P < 0.001.

Because BBR could limit the expansion of both growing and mature CRC organoids, we wonder how BBR exhibits such an antitumor activity in the organoids. Firstly, we examined the cell proliferation capacity in KPC organoids after 36 hours of BBR treatment. It was demonstrated that there were less Ki67 and PCNA positive cells within BBR-treated KPC organoids compared with the control KPC organoids as shown by immunohistochemical (IHC) staining (**Figure 4A**). Statistical results showed that the ratio of Ki67^+^ and PCNA^+^ cells in each KPC organoids was also significantly decreased due to BBR treatment (**Figures 4B, C**). DNA replication is essential to the successful growth of organoids, so we further evaluated this activity using EdU incorporation assay. The *in situ* EdU staining results clearly demonstrated that BBR caused a dose-dependent decreasing of EdU-positive cells within both KPC and Caco-2 organoids (**Figures 4D, F**). And the quantitative analysis also confirmed this tendency (**Figures 4E, G**). These results indicate that BBR could strongly blocks the cell proliferation and limits DNA replication of CRC organoids.

### 3.5 BBR generates lethal mitochondria-damaging ROS in CRC organoids

**Figure 5:**
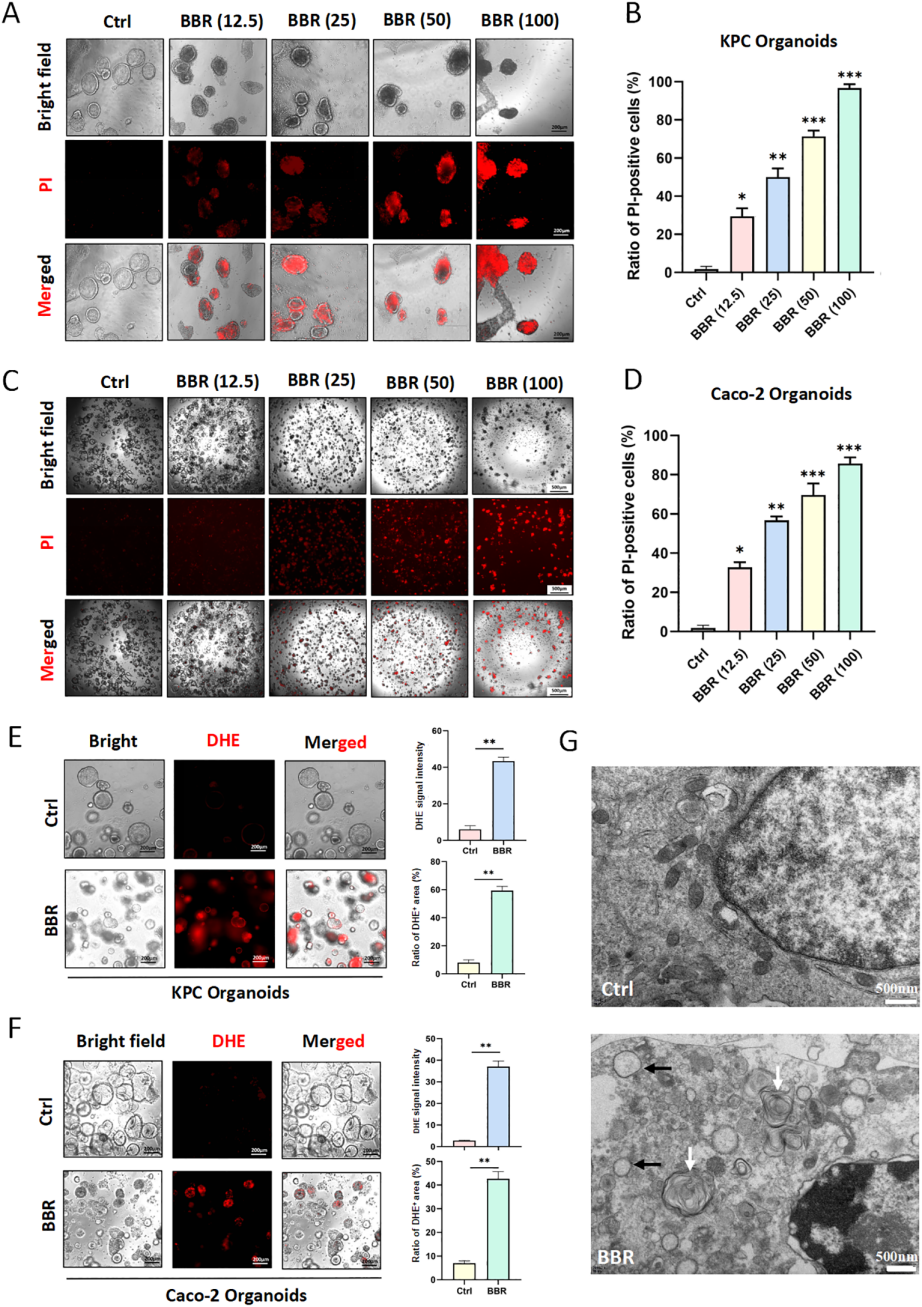
Berberine induces cell death and reactive oxygen species (ROS) generation in KPC and Caco-2 organoids. **(A)** Representative bright field (Trans), PI staining (dead cells, red), and merged images of KPC organoids treated with BBR at the indicated concentrations (12.5, 25, 50, 100 μg/mL) for 24 hours. Scale bar, 200 μm. **(B)** Quantitative analysis for the percentage of PI-positive cells in KPC organoids in **(A)**. **(C)** Representative bright-field, PI staining, and merged images of Caco-2 organoids at 24-hour after the treatment of indicated BBR concentrations. Scale bar, 500 μm. **(D)** Statistical analysis for the ratio of PI-positive cells in Caco-2 organoids. **(E)** Representative images of DHE staining in KPC organoids to show ROS content after 24 hours of 25 μg/mL BBR treatment. Quantitative results showed the intensity of DHE signals and the ratio of DHE^+^ area. Scale bar, 200 μm. **(F)** The images of DHE staining results in Caco-2 organoids after 24-hour BBR treatment (25 μg/mL). The intensity of DHE signals and ratio of DHE^+^ area was significantly increased compared with the control Caco-2 organoids. Scale bar, 200 μm. **(G)** Representative TEM images of control and BBR-treated KPC organoids. The black arrows indicated swelling mitochondria with diminishing cristae, and the white arrows showed the mitochondria undergoing mitophagy. Scale bar, 500 nm. *P < 0.05, **P < 0.01, ***P < 0.001.

To further characterize the biological effects of BBR in CRC organoids, we examined cell death and reactive oxygen species (ROS) levels in KPC and Caco-2 organoids. PI staining revealed that the number of PI-positive dead cells was gradually elevated in both organoid models with increasing concentrations of BBR (**Figures 5A, C**), accompanied by a significant reduction in organoid size as previously observed. Quantitative analysis verified a marked increase in the percentage of PI-positive cells (**Figures 5B, D**). In addition, intracellular ROS levels were detected using DHE fluorescent probe. Compared with the control group, treatment with 25 μg/mL BBR significantly enhanced the red fluorescence intensity of DHE in both KPC and Caco-2 organoids, demonstrating that BBR notably induced ROS overproduction (**Figures 5E, F**). Additionally, other doses of BBR treatments also had the identical results in both KPC organoids and Caco-2 organoids (**Supplementary Figures S3A, B**). Moreover, in order to confirm the mitochondrial damage caused by BBR treatment, we examined the ultrastructure of KPC organoids using transmission electron microscopy (TEM). It was observed that in BBR-treated organoids, the nucleus chromatin became more condensed, and the cristae structure was severely compromised. Importantly, there were more round swelling mitochondria (black arrows) and autophagic mitochondria (white arrows) in the cytoplasm as compared to the control KPC organoids (**Figure 5G**, **Supplementary Figure S5A**). Therefore, these results indicate that BBR treatment induces significant structural and functional changes in mitochondria and increases ROS content to cause cell death in CRC organoids.

### 3.6 BBR treatment causes significant cell cycle arrest in CRC organoids

**Figure 6:**
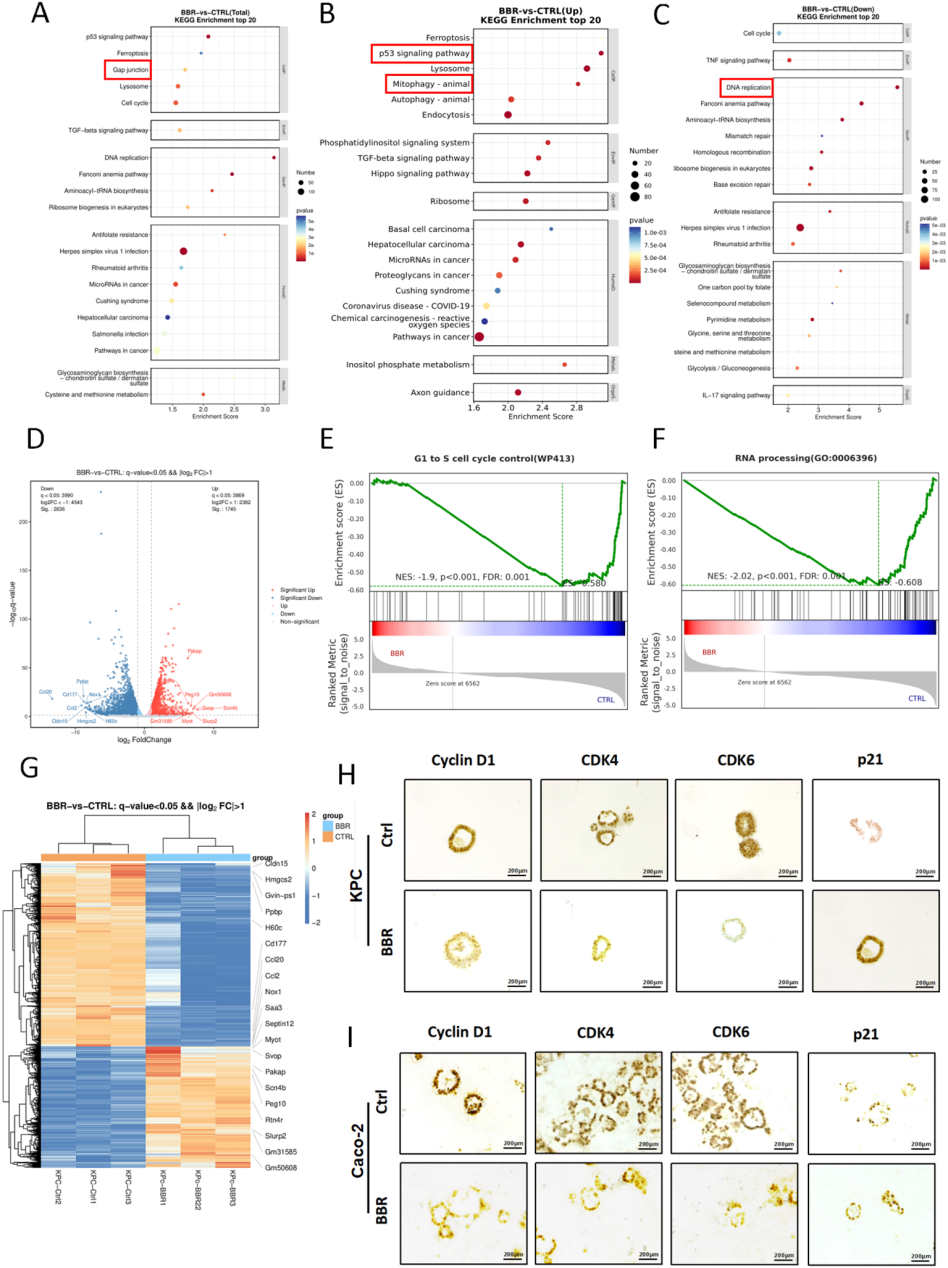
The integrated analysis of RNA-Seq assays and validation of cell cycle arrest proteins after BBR treatment. **(A)** KEGG pathway enrichment analysis showed the related top 20 pathways of those changed genes among the total DEGs in BBR-treated KPC organoids *vs.* control KPC organoids. **(B)** Top 20 KEGG pathway enrichment pathways of upregulated genes in the BBR-treated group compared to the control group. **(C)** The top 20 KEGG pathway enrichments of downregulated genes after BBR treatment as compared to the control KPC organoids. **(D)** Volcano plot of DEGs in KPC organoids after BBR treatment in KPC organoids. Screening threshold: false discovery rate (FDR, q) < 0.05 and |log_2_(fold change)| ≥ 1. Significantly up- and down-regulated genes are marked in red and blue, respectively. **(E)** Heatmap of the most significantly changed genes in KPC organoids at 24h after 25 μg/mL BBR treatment. **(F)** Gene Set Enrichment Analysis (GSEA) of the genes involving G1-to-S cell cycle control (WP413). **(G)** GSEA results of those genes involving RNA processing (GO:0006396) pathway. **(H)** Immunohistochemical (IHC) validation for the protein expression changes of in key cell cycle proteins, including Cyclin D1, CDK4, CDK6, and p21, between the control KPC organoids and BBR-treated KPC group. Scale bar: 200 μm. **(I)** IHC validation of the level of Cyclin D1, CDK4, CDK6, and p21 in BBR-treated Caco-2 organoids against the control Caco-2 organoids. Scale bar: 200 μm.

In order to systematically elucidate the anti-tumor molecular mechanisms of BBR in CRC organoids, we subsequently performed RNA-Seq assay, and an integrated analysis of the transcriptional reprogramming induced by BBR. KPC organoids after 24-hour treatment of 25 μg/mL BBR and the control KPC organoids were used for RNA-Seq assay. Differential expression gene (DEG) analysis revealed that BBR treatment triggered an extensive transcriptional reprogramming, including 1,745 upregulated DEGs and 2,636 downregulated DEGs (**Supplementary Figure S4A**). Principal component analysis (PCA) further confirmed the significant separation of BBR-treated KPC organoids from the control KPC organoids (**Supplementary Figure S4B**), indicating BBR induced a highly consistent transcriptional response. KEGG pathway enrichment analysis identified that BBR induced pathways changes in gap junction, DNA replication, p53 signaling, mitophagy, etc (**Figures 6A-C**). And Wikipathways enrichment results demonstrated that G1 to S cell cycle control, triacylglyceride and other metabolic pathways are greatly changed after BBR treatment (**Supplementary Figures S4C-E**). Interestingly, the volcano plot visually demonstrated that Hmgcs2, Nox1, and other chemokine-related genes were significantly decreased after BBR treatment (**Figure 6D**), and these critically changed functional genes were individually listed in the heatmap (**Figure 6E**). Using GSEA analysis, it was confirmed that the genes involving G1 to S cell cycle control and RNA processing were significantly changed due to BBR treatment (**Figures 6F, G**). Moreover, other genes controlling important biological functions were also changed, including tissue development, cytoplasmic translation, translation at presynapse (**Supplementary Figure S4F-H**). Importantly, the critical cell cycle control findings were independently validated at the protein level via immunohistochemical (IHC) staining using control and BBR-treated organoid sections. The staining results clearly showed significantly differential positive signals in the level of Cyclin D1, CDK4, CDK6, and p21 among KPC organoids (**Figure 6H**) and Caco-2 organoids (**Figure 6I**) respectively. In summary, these data integrate RNA-Seq and IHC evidence to demonstrate that BBR treatment achieves stable cell cycle arrest at the functional gene and the protein contents to block the expansion of CRC organoids, providing molecular evidence for its capacity in the therapeutic use of colorectal cancer.

### 3.7 BBR disrupts the tight junction and epithelial integrity in CRC organoids

**Figure 7:**
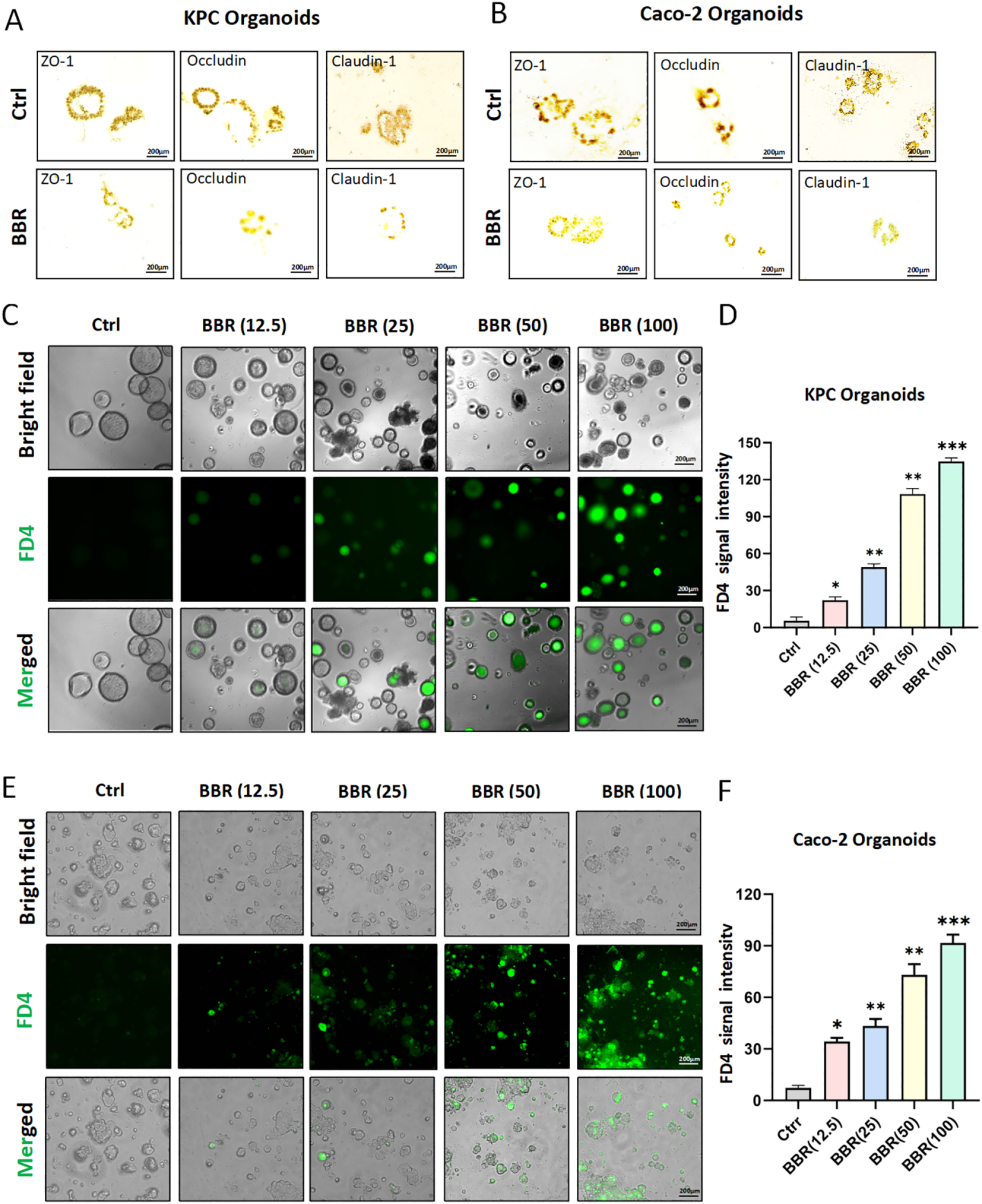
BBR decreases the expression of tight junction protein and induces an increasing of epithelial permeability in CRC organoids. **(A, B)** Representative IHC staining images of ZO-1, Occludin, and Claudin-1 in KPC organoids **(A)** and Caco-2 organoids **(B)** between control group and BBR-treated group. Scale bar = 200 μm. **(C)** FITC-dextran (FD4, green) assay revealed an increased organoid permeability at 24h after BBR treatment (12.5μg/mL, 25μg/mL, 50μg/mL, and 100 μg/mL) compared with control KPC organoids. Scale bar = 200 μm. **(D)** Quantitative analysis for the intensity of FD4 dye within KPC organoids. **(E)** FD4 staining of Caco-2 organoids with or without the same BBR treatment. Scale bar = 200 μm. **(F)** Statistical results for the FD4 signal intensity within Caco-2 organoids among different group. *P < 0.05, **P < 0.01, ***P < 0.001.

According to the implication of the above RNA-Seq data, we further examined BBR’s effects on the epithelial barrier function of CRC organoids. Firstly, we detected the tight junction proteins in KPC and Caco-2 organoids. The IHC results evidenced that BBR treatment apparently decreased the staining intensity of ZO-1, Occludin, and Claudin-1 in these two CRC organoids (**Figures 7A, B**), suggesting that BBR effectively disrupts the tight junction proteins to induce organoid damage. To further evaluate the functional consequences of BBR on epithelial integrity alterations, we employed a FITC-labeled dextran (FD4) leakage assay to examine the paracellular permeability. In KPC organoids, FD4 fluorescence intensity progressively enhanced with the increasing of BBR’s concentrations (**Figure 7C**). Quantitative analysis revealed that the FD4-positive intensity within each group of BBR-treated KPC organoids was significantly higher than the control group, demonstrating a clear dose-dependent effect (**Figure 7D**). Highly consistent results were also obtained in Caco-2 organoids after gradient BBR treatment, and FD4 fluorescence intensity was increased in a gradient with rising BBR concentrations (**Figure 7E**). Statistical analysis showed higher FD4 intensity within Caco-2 organoids after BBR treatment (**Figure 7F**). Collectively, our data clearly demonstrated that while BBR specifically disrupts the tight junction proteins, it functionally increases the paracellular permeability and induces the breakdown of epithelial integrity of CRC organoids.

### 3.8 BBR inhibits lipid accumulation in CRC organoids via blocking fatty acid synthesis

**Figure 8:**
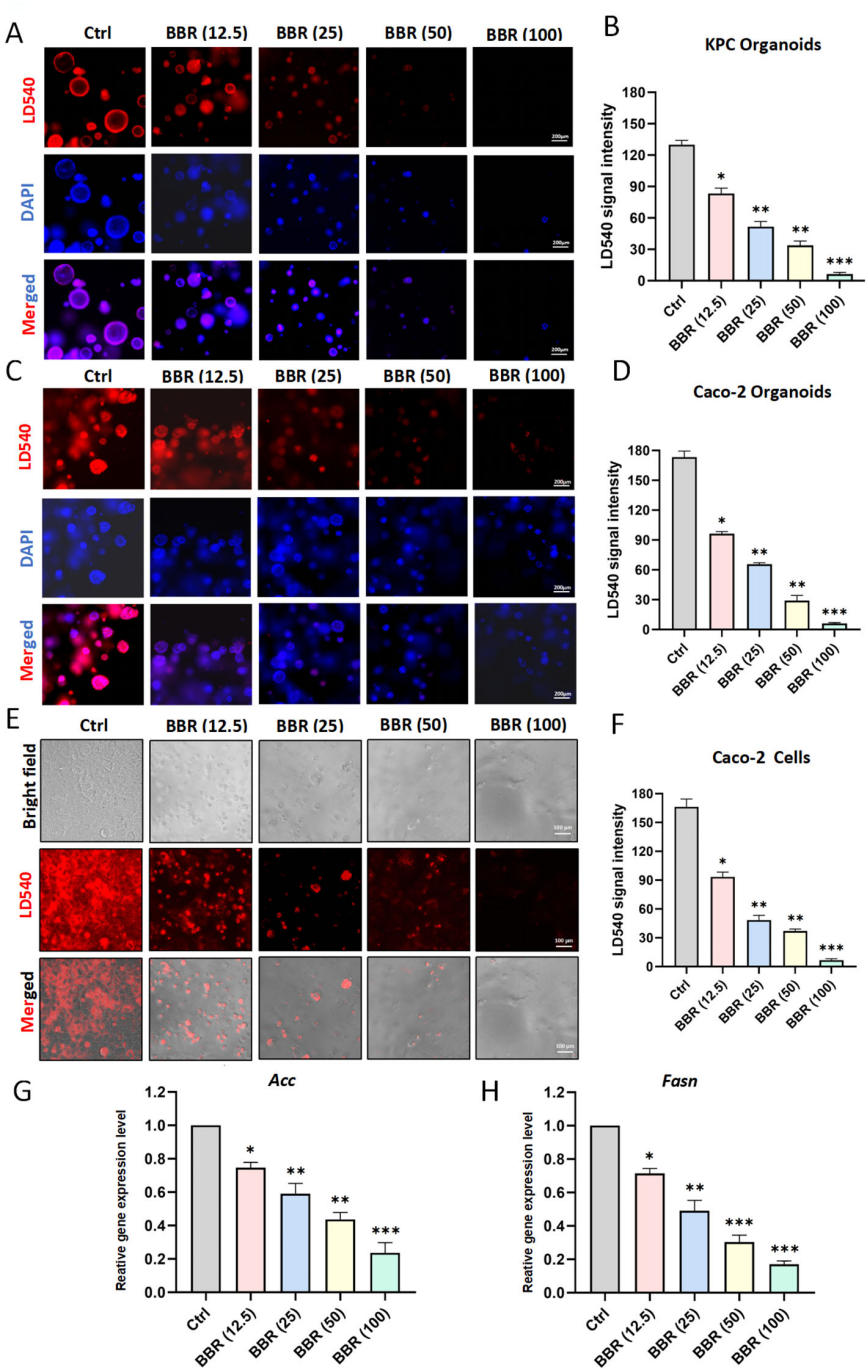
BBR reduces lipid accumulation and suppresses the expression level of lipid synthesis related genes in CRC organoids and cells. **(A)** Representative fluorescent images of LD540 (lipophilic dye) staining in KPC organoids at 24h after the different doses of BBR treatment (0, 12.5, 25, 50, and 100 μg/mL). Scale bar: 200 μm. **(B)** Quantitative analysis of LD540 signal intensity in KPC organoids of different groups. **(C)** Fluorescent images of LD540 staining in Caco-2 organoids at 24h after the same BBR treatment described in **(A)**. Scale bar: 200 μm. **(D)** Quantitative analysis of LD540 signal intensity in Caco-2 organoids of different groups as compared to the control group. **(E)** Representative LD540 staining images of Caco-2 cells at 24h after different BBR treatments (0, 12.5, 25, 50, and 100 μg/mL). Scale bar: 100 μm. **(F)** Statistical analysis for the mean LD540 signal intensity of Caco-2 cells in each group. **(G)** qRT-PCR examination results for the gene expression level of *Acc* in KPC organoids after the different dose treatments of BBR. **(H)** The results of qRT-PCR examination for *Fasn* gene expression of KPC organoids after BBR treatments. *P < 0.05, **P < 0.01, ***P < 0.001 compared to the control group.

**Figure 9:**
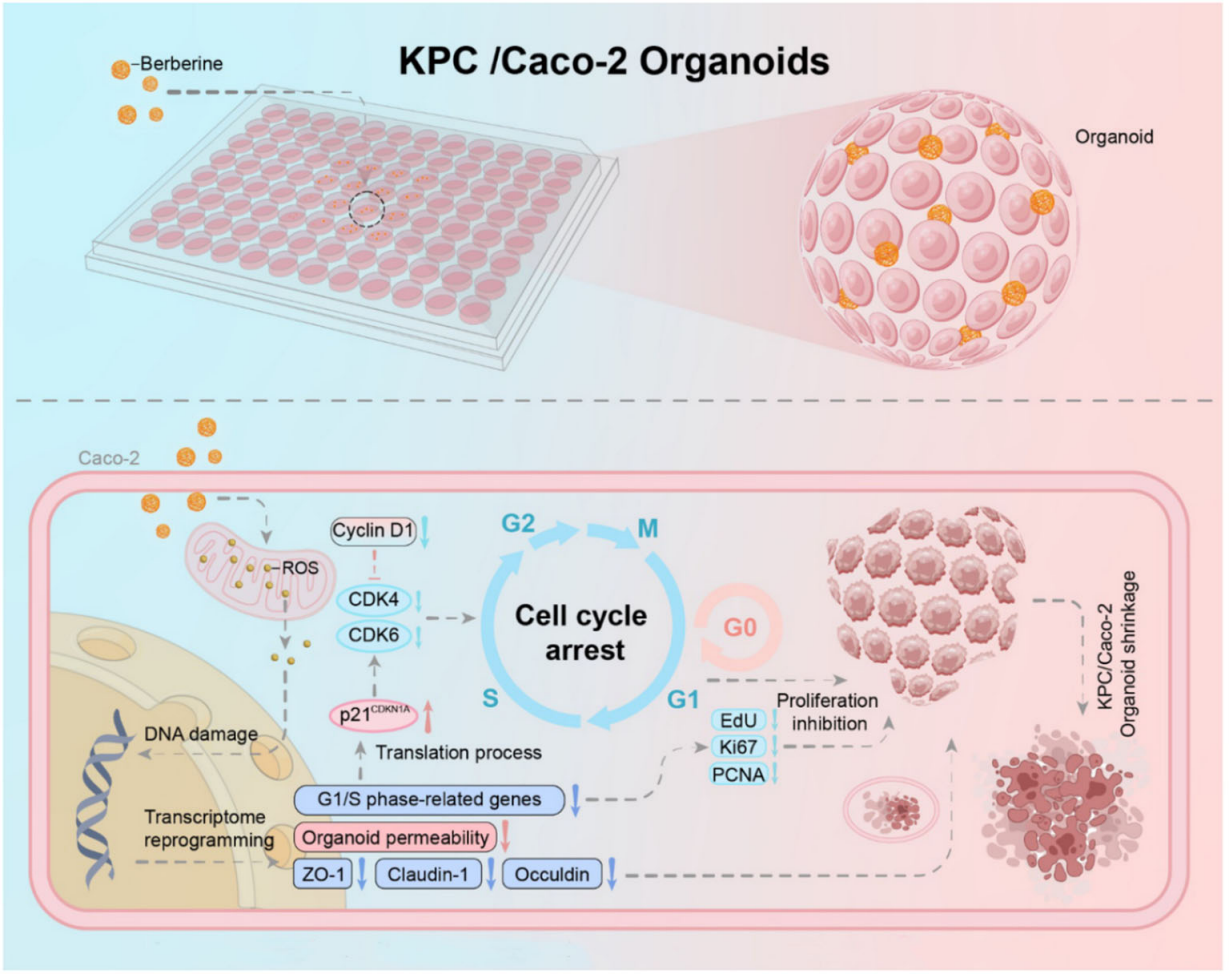
Schematic illustration shows berberine (BBR) could restrain the expansion of CRC organoids by blocking cell cycle progression and reducing lipid synthesis.

High level lipid metabolism is a key characteristic of colorectal cancer and other cancer types because fatty acid provides necessary fuel for the rapid expansion of cancer cells. Due to the RNA-Seq results, we also investigated the influences of BBR on the lipid metabolism in CRC organoids. LD540 is a highly sensitive lipid probe with red fluorescence, so we used it to stain the lipid within CRC organoids cells. lipid probes. It was clearly shown that KPC organoids without BBR treatment had a strong LD540 signals surrounding the organoids, but with the increasing doses of BBR the red fluorescent signals were dramatically decreased at 24h (**Figure 8A**). Quantitative analysis also revealed such a descending of lipid accumulation within KPC organoids (**Figure 8B**). When we repeated this assay using human Cac-2 organoids, the same images and statistical analysis were demonstrated after BBR treatment (**Figures 8C, D**). To exclude the 3D spatial influences on the observation of LD540 signals, we further performed the staining on 2D cultured Caco-2 cells. Similarly, the high LD540-positive signals flat Caco-2 cells were gradually decreased with the increasing of BBR concentration (**Figure 8E**). And statistical results demonstrated a significant decaying of LD 540 signal intensity (**Figure 8F**). Finally, we also validated the mRNA expression levels of genes regulating lipid accumulation. *Acetyl-CoA carboxylase alpha (Acc)* and *fatty acid synthase (Fasn)* are two essential genes during intracellular lipid synthesis. We performed qRT-PCR assay to examine the changes of their mRNA level within KPC organoids after the loading of BBR. mRNA expression of *Acc* and *Fasn* after BBR treatment was significantly less compared with their levels within control KPC organoids (**Figures 8G, H**). So, these findings indicate that BBR is a strong component of traditional Chinese medicine, which can downregulate the essential gene expression level during fatty acid synthesis in CRC organoids (**Figure 9**).

## 4 Discussion

Today, colorectal cancer (CRC) remains one of the leading types of gastrointestinal malignancies. Although many efforts and progresses have reported recently concerning its basic research and clinical treatment, there are still many unresolving obstacles, such as chemotherapy resistance, severe side effects, potential recurrence risks, and a low therapeutic index (26). So, there is an urgent need to explore new adjuvant therapies and alternative treatments for colon cancer and other malignant tumors. Among all the emerging candidates, traditional Chinese medicinal herbs and their extracts have attracted more attentions and interests of both scientists and clinicians, so it is of great worth to excavate and validate the exact biological functions of traditional and novel components of medicinal herbs, driving the search for effective phytotherapeutic approaches (27–30).

*Coptis chinensis* is a versatile Chinese medicinal herb with a long use history, and berberine (BBR) has been recognized as its major active biological component. We and other researchers have identified multiple new functions and mechanisms of BBR in gastrointestinal diseases. For example, BBR benefits the homeostasis of intestinal epithelium and its reconstitution after radiation injury (5), and it could regulate the submandibular gland function to alleviate radiation-induced xerostomia in mice (6). At the beginning of this work, we have also performed the safety assessment of BBR on the healthy colon tissues and organoids. As expected, BBR exhibits no detectable negative impairments on them (**Figure 1**). In the CRC research, BBR is capable to modulate the gut microbiota and immune cells, such as macrophages, T cells, and neutrophils. Although the direct influences of BBR on CRC cells have been investigated before, most of those related experiments are performed using 2D cultured monolayer CRC cell lines, which don’t have 3D spatial characteristics thus represent less real response of *in vivo* CRC cells after BBR treatment. Clinical studies have demonstrated the therapeutic effects of BBR against colorectal adenoma recurrence persisted for up to 6 years after the 2-year intervention period (31). The potent proliferation inhibition observed in our study offers critical *ex vivo* mechanistic support for this sustained clinical effect.

Organoids offer a more physiologically relevant model, capturing 3D spatial characteristics and key features of the native tumor microenvironment. In this study, we have established CRC organoid models, including both genetically engineered KPC mouse organoids and human Caco-2 cell line-derived sphere organoids. And then we systematically investigate BBR’s anti-tumor effects using multiple approaches. We have confirmed that BBR significantly inhibits the proliferation of CRC organoids, inducing G1/S cell cycle arrest, which aligns with the prior reports. Furthermore, we have identified oxidative stress, which is triggered by mitochondrial ROS accumulation, as a key driver of BBR-induced cell death, which is consistent to the previous report (32). Beyond BBR’s direct cytotoxicity, our study also highlights its dual roles in breaking the epithelial barrier of CRC organoids. We show that BBR impairs tight junction integrity in CRC organoids by downregulating ZO-1, Occludin, and Claudin-1, a finding with implications for tumor dissemination and the gut barrier. Actually, BBR has been reported to protect the integrity of colonic epithelium in DSS-induced colitis model. Therefore, we have also isolated healthy colonic crypts (**Supplementary Figure S5B**) and tested whether BBR could harm healthy colonic organoids. Interestingly, we find that different doses of BBR (ranging from 12.5-100 μg/mL gradients) could not disrupt the permeability of these organoids (**Supplementary Figure S5C**). Therefore, it seems that BBR exhibits differential influences on healthy and cancerous organoids.

While BBR restrains the cell cycle progression of CRC organoids, we find a significant accumulation of ROS, which might be caused by the damaged mitochondria (**Figure 5G**; **Supplementary Figure S5A**). According to the established knowledge, arrested cell cycle and increased ROS can lead to cellular apoptosis. BBR induces ROS production via the mitochondrial pathway, causes serious structural and functional damage to mitochondria, and ultimately leads to CRC cell apoptosis. During this study, we find BBR could interrupt the permeability of CRC organoids as shown by increased FD4 signal after BBR treatment, and we wonder whether BBR could harm the integrity of healthy colonic organoids, since BBR has reported to protect the epithelial integrity in DSS-induced experimental colitis (33). Intriguingly, our results show BBR could not result FD4 leakage in the normal colonic organoids (**Supplementary Figure S5C**). Importantly, when we use Z-VAD to block the apoptosis, the FD4 dye leaked into KPC organoids was apparently decreased, indicating the damaged epithelial integrity is a consequence due to induced cell death (**Supplementary Figure S5D**). Therefore, we conclude that BBR could differentially influence the CRC organoids and healthy colonic organoids.

Our transcriptome sequencing analysis also reveals that BBR induces extensive gene expression reprogramming, and DEGs are significantly enriched in gap junction, p53 signaling pathway, mitophagy, DNA replication, and adipogenesis genes. Recent studies show that BBR significantly activates the p53 signaling pathway in CRC cells while systematically downregulating genes associated with DNA replication and cell cycle progression (34–36), and BBR is able to promote LC3-II/I ratio of SW480 cells in a dose dependent manner (33). The enrichment of autophagy pathways may reflect BBR’s fine-tuned regulation of cellular fate in CRC organoids. Meanwhile, both our results and the previous reports have shown a high level of lipid metabolism. We find BBR could inhibit lipid accumulation within CRC organoids and Caco-2 cells, and the impaired mRNA expression of *Acc* and *Fasn* might be responsible for these effects, which are required for lipid synthesis.

In the present study, there are still several limitations. First of all, despite tumor organoid is a powerful tool, our CRC model lacks the full complexity of tumor microenvironment (TME), including immune cells and stromal cells, such as tumor associated macrophages (TAMs) and cancer-associated fibroblasts (CAFs) (37, 38). Since TAMs and CAFs can secrete inhibitory cytokines, such as TGF-β and IL-10, thus creating an immunosuppressive milieu that dampens anti-tumor immunity and promotes treatment resistance (39, 40). Some previous studies have demonstrated that BBR suppresses PD-L1 expression to activate T cells in lung cancer (41) and modulates macrophage-mediated tumor cell death in melanoma (42). However, here we have only observed BBR’s direct impairment on CRC organoids without introducing any immune cells. Our future research may further explore BBR’s effects on the combination of CRC organoids and immune cells and the potential mechanisms. Secondly, BBR could regulate intestinal barrier function via modulating gut microbiota composition, increasing the abundance of short-chain fatty acid-producing bacteria such as *Roseburia* and *Faecalibacterium* (36). The current CRC organoid study could still not answer the gut microbiota mediated antitumor effects of BBR. Thirdly, here we mainly used CRC organoids from KPC mice, and we are seeking to perform the culture of cancer organoids using clinical CRC tissues from patient-derived samples. Finally, although transcriptomic analysis revealed multiple potential pathways, their causal relationships require further validation via gene knockout/overexpression approaches.

## 5 Conclusions

This study demonstrates that BBR is a strong herbal compound which suppresses the expansion of CRC organoids by inducing oxidative stress and cell cycle arrest, reprogramming many critical transcriptomic networks, and notably enhancing the epithelial permeability of CRC organoids. These results provide new experimental evidence supporting recent clinical data, and this research also highlight BBR’s dual “tumor-suppressive and gut-protective” potential during the management of gastrointestinal health of CRC patients. As a safe, inexpensive, and readily available natural compound, BBR shows a great promise in the chemoprevention and adjuvant therapy of colorectal cancer. Future research should focus on *in vivo* efficacy validation, optimization of combination therapy strategies, and in-depth exploration of its mechanisms based on gut microbiota regulation.

## Data Availability

All the original data included in the article are available upon reasonable request to the corresponding authors.
