# Supplementary material for: Berberine restrains the expansion of colorectal cancer organoids by blocking cell cycle progression and reducing lipid synthesis
Source: Front Immunol. 2026 Jul 10;17:1855151. doi: 10.3389/fimmu.2026.1855151 (PMC13395616; doi:10.3389/fimmu.2026.1855151)

**Supplementary materials**

**Figure legend**

**Figure S1. Screening results for the influences of different BBR doses on the growth of various CRC organoids.** (A) The growth inhibition of BBR at 12.5 μg/mL, 50 μg/mL, and 100 μg/mL on KPC organoids at the early stage. Scale bar: 500 μm. (B) BBR at the doses of 12.5 μg/mL, 50 μg/mL, and 100 μg/mL also exhibited an inhibition on the growth of Caco-2 organoids. Scale bar: 500 μm. (C) HCT-116 organoids also showed an inhibition response to the BBR treatment. Scale bar: 500 μm.

**Figure S2. Different doses of BBR treatment significantly impair the expansion of mature CRC organoids.** (A) The size of mature KPC organoids at Day 2 and Day 3 was dramatically decreased after the loading of different BBR doses (12.5 μg/mL, 50 μg/mL, and 100 μg/mL) as compared to the control KPC organoids. Scale bar: 500 μm. (B) Different BBR doses, including 12.5 μg/mL, 50 μg/mL, and 100 μg/mL, significantly decreased the size of mature Caco-2 organoids at the indicated time points. Scale bar: 500 μm.

**Figure S3. BBR treatment causes an increasing level of ROS content within CRC organoids.** (A) Different doses of BBR gradually increased the ROS level in KPC organoids as indicated by DHE staining. Scale bar: 200 μm. (B) Different BBR doses, including 12.5 μg/mL, 50 μg/mL, and 100 μg/mL, significantly increased the ROS level stained by DHE probes in Caco-2 organoids. Scale bar: 500 μm.

**Figure S4. RNA-Seq results for KPC organoids in the control group *vs.* BBR-treated group.** (A) There were 1,745 upregulated and 2636 downregulated DEGs in BBR-treated KPC organoids as compared to the control KPC organoids. (B) PCA analysis for the RNA-Seq data revealed an apparent separation between BBR-treated KPC organoids and their control group. (C,D,E) Wikipathway enrichment of top 20 total DEGs (C), upregulated DEGs (D), and down regulated DEGs (E) in BBR-treated KPC organoids as compared to the control KPC organoids. Cell cycle control and lipid metabolism are the two critical changed pathways after BBR treatment. (F,G,H) GSEA analysis of DEGs involved in tissue development (F), cytoplasmic translation (G), and transition at presynapse (H).

**Figure S5. Effect of BBR on KPC mitochondria, healthy colonic organoids, and the combined treatment with Z-VAD on KPC organoids.** (A) Higher magnification of damaged mitochondria in KPC organoids shown in Fig. 5G. (B) Representative image of freshly colonic crypts isolated from healthy C57BL/6 mouse before the organoid culture. Scale bar: 500 μm. (C) Bright-field, FD4 fluorescent channel and composite images of healthy colonic organoids at 24 h after the treatment with different doses of BBR from 12.5 μg/mL, 25 μg/mL, 50 μg/mL, and 100 μg/mL. Scale bar: 200 μm. (D) The apoptosis inhibitor Z-VAD attenuated BBR-induced leakage of FD4 into KPC organoids. Scale bar, 200 μm.

**Table S1. The information of Antibodies used in this study**

| **Antibody** | **Provider** | **Catalog** | **Host species** | **Dilution** |
| --- | --- | --- | --- | --- |
| CDK4 | Cell Signaling | 12790 | Rabbit | 1:400 |
| CDK6 | Cell Signaling | 3136 | Mouse | 1:400 |
| Claudin-1 | Proteintech | 13050-1-AP | Rabbit | 1:200 |
| Cyclin D1 | Cell Signaling | 2978 | Rabbit | 1:400 |
| Ki67 | Abcam | ab16667 | Rabbit | 1:500 |
| Occludin | Proteintech | 13409-1-AP | Rabbit | 1:200 |
| PCNA | Abcam | ab92552 | Rabbit | 1:400 |
| p21 | Cell Signaling | 2947 | Rabbit | 1:400 |
| ZO-1 | Proteintech | 21773-1-AP | Rabbit | 1:200 |

**Table S2. Primer sequences used for qRT-PCR assay**

| **Gene** | **ID** | **Forward primer** | **Reverse primer** |
| --- | --- | --- | --- |
| Actb | 11461 | CTTCTTTGCAGCTCCTTCGTT | TTCTGACCCATTCCCACCA |
| MAcc | 107476 | CCCGTGAGAACACAGAGATAAA | CTGAGGTGGTTGAGTGTGTT |
| Fasn | 14104 | GGAGGTGGTGATAGCCGGTAT | TGGGTAATCCATAGAGCCCAG |


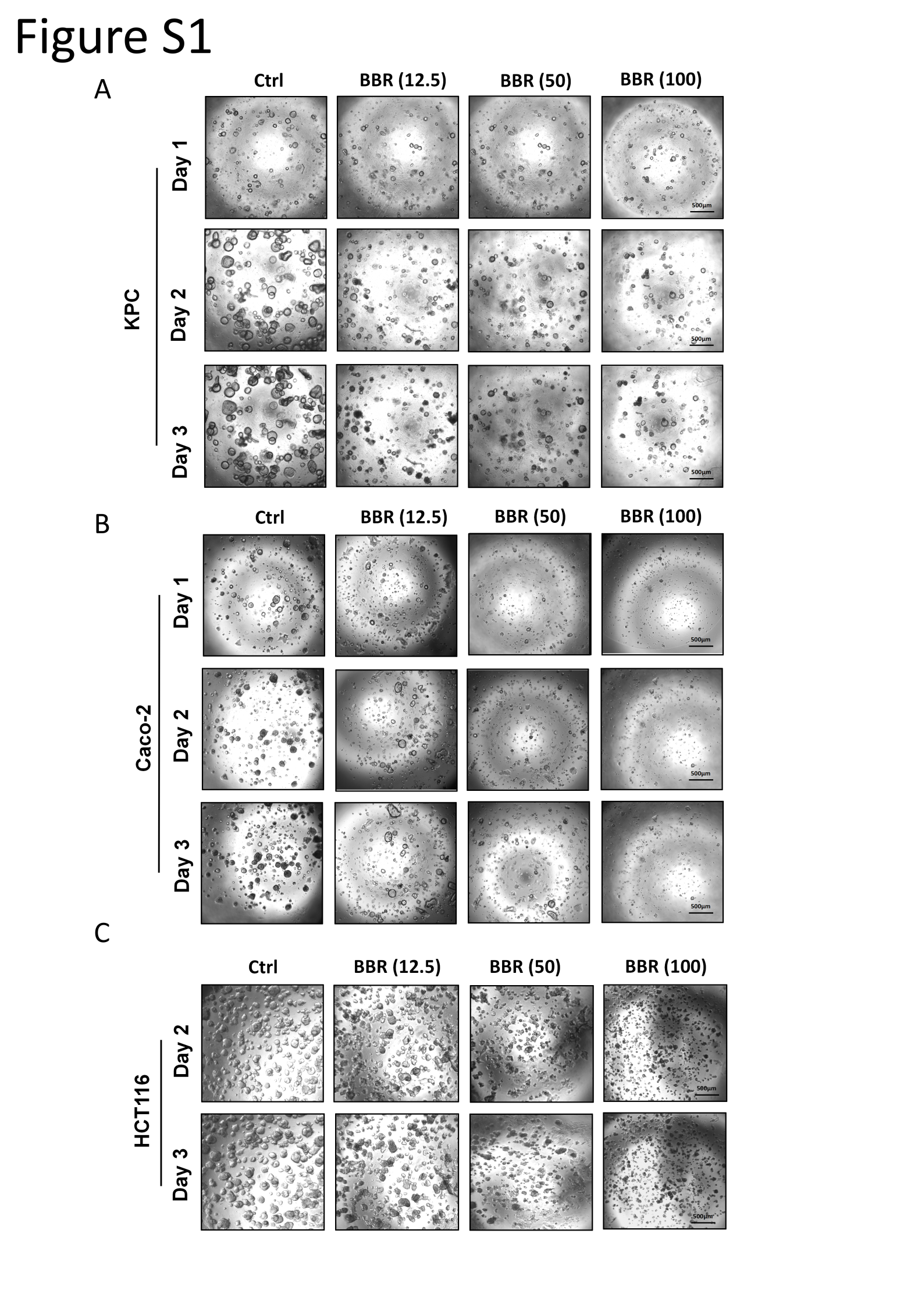


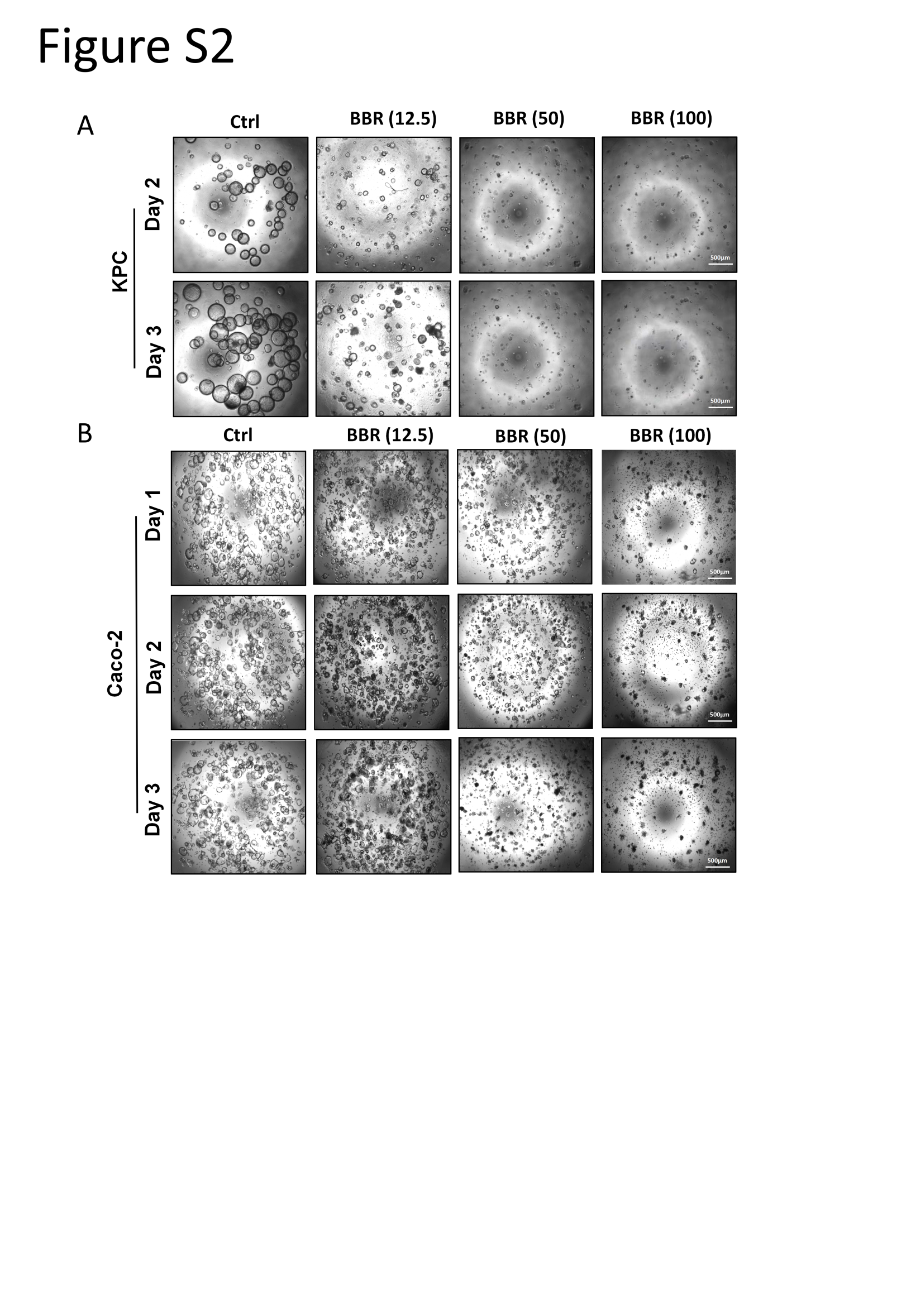


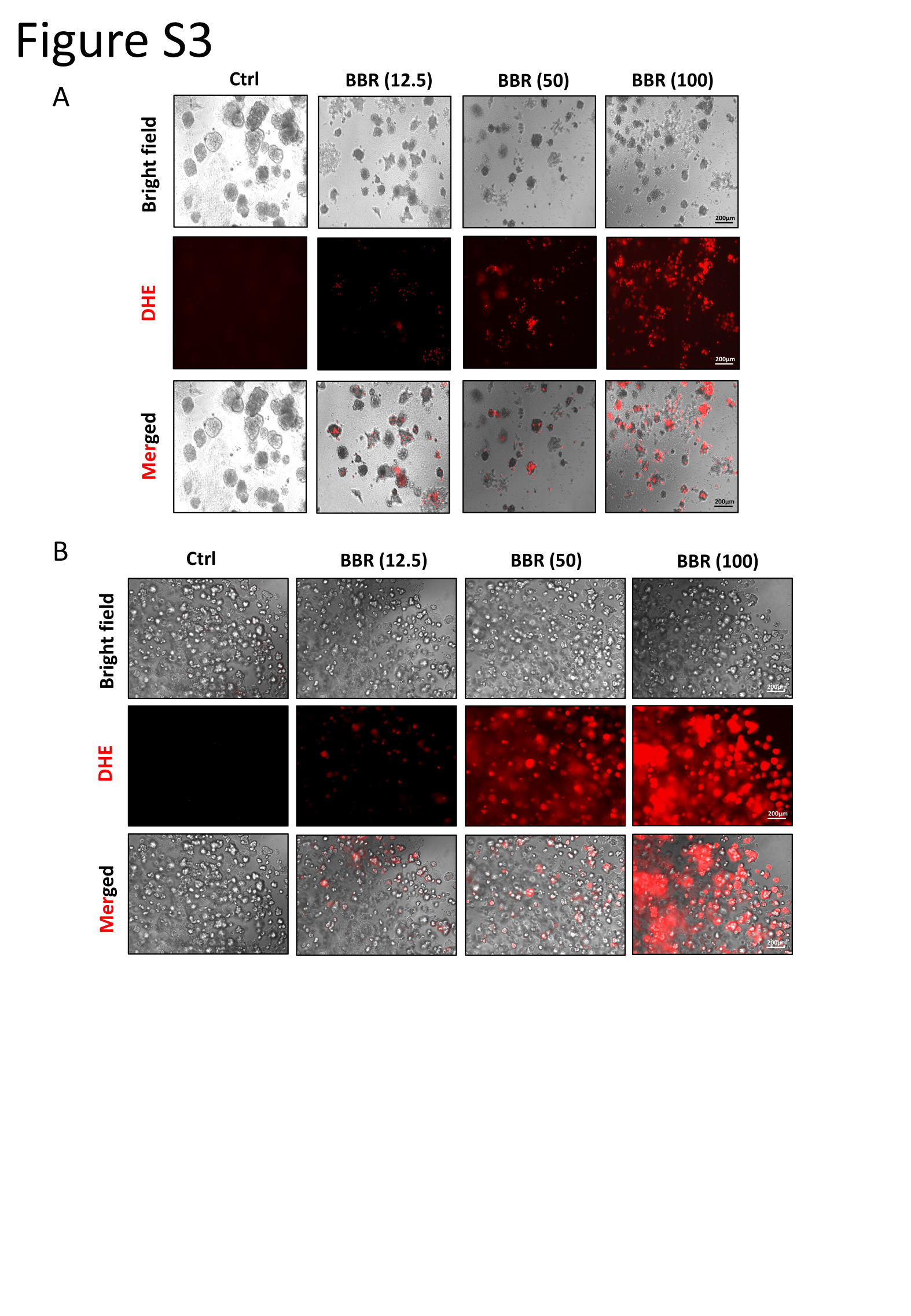

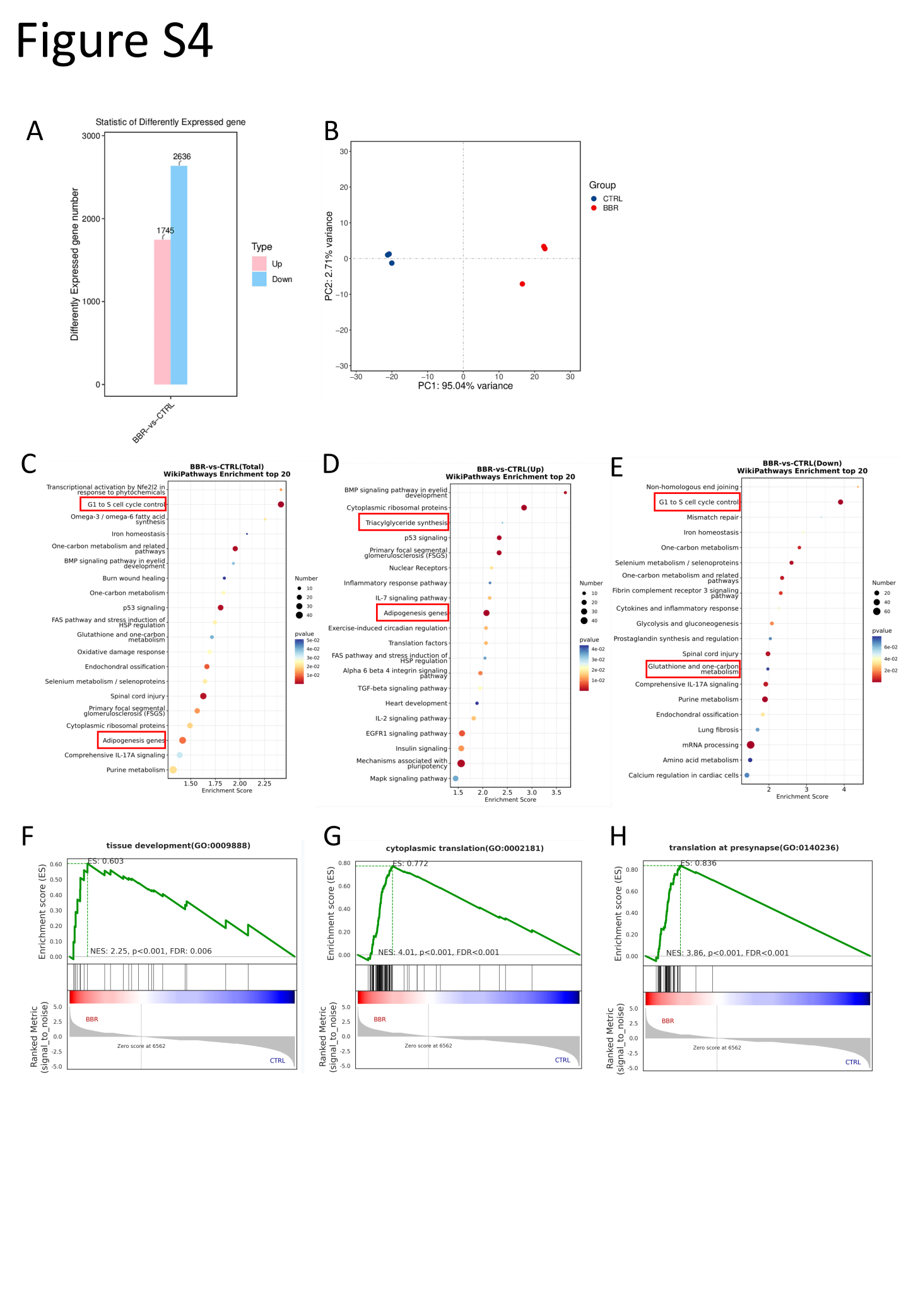


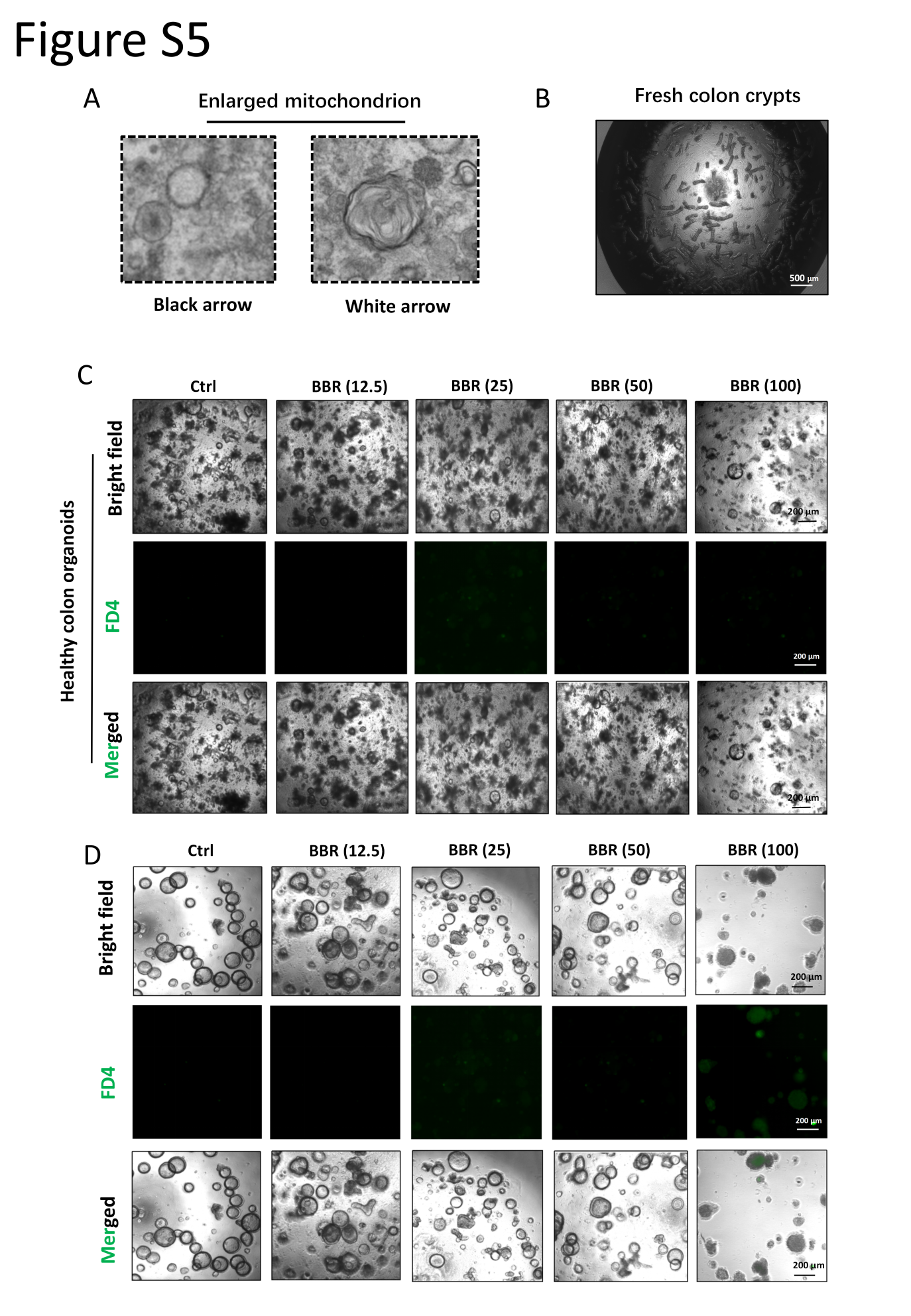

Supplement: Supplementary file 1 [file SupplementaryFile1.docx]
